# Oncology Biomarkers, Clinical Characteristics, and Survival Outcomes in Colorectal Cancer Patients with Spinal Metastases Undergoing Spinal Surgery: Insights from a Retrospective Cohort Study

**DOI:** 10.3390/cancers17111739

**Published:** 2025-05-22

**Authors:** Abdel-Hameed Al-Mistarehi, Taha Khalilullah, Abdul Karim Ghaith, Mahnoor Shafi, Jawad M. Khalifeh, Yuanxuan Xia, Khaled J. Zaitoun, Ahmad A. Alnasser, Joseph Rajasekaran, Avi N. Albert, Siddharth Shah, Nicholas Theodore, Jeffrey Meyer, Kristin J. Redmond, Susan L. Gearhart, Daniel Lubelski

**Affiliations:** 1 Department of Neurosurgery, School of Medicine, Johns Hopkins University, Baltimore, MD 21205, USA; aalmist1@jh.edu (A.-H.A.-M.); tkhalil1@jh.edu (T.K.); aghaith1@jh.edu (A.K.G.); mshafi5@jh.edu (M.S.); jkhalif1@jhmi.edu (J.M.K.); kjzaitoun20@med.just.edu.jo (K.J.Z.); aalnass3@jh.edu (A.A.A.); aalbert24@mmc.edu (A.N.A.); theodore@jhmi.edu (N.T.); 2 Department of Neurosurgery, Drexel University College of Medicine, Philadelphia, PA 19129, USA; jvr38@drexel.edu; 3 School of Medicine, Meharry Medical College, Nashville, TN 37208, USA; 4 Department of Neurosurgery, RCSM Government Medxical College, Kolhapur 416003, India; siddharth.dr99@gmail.com; 5 Department of Radiation Oncology & Molecular Radiation Sciences, School of Medicine, Johns Hopkins University, Baltimore, MD 21205, USA; jmeyer58@jhmi.edu (J.M.); kjanson3@jhmi.edu (K.J.R.); 6 Department of Surgery, Division of Colorectal Surgery, School of Medicine, Johns Hopkins University, Baltimore, MD 21287, USA; sdemees1@jhmi.edu

**Keywords:** colorectal cancer, spinal metastases, biomarkers, CK20, survival, surgical intervention

## Abstract

**Objectives:** This study aims to identify clinical characteristics and biomarkers influencing survival outcomes in colorectal cancer (CRC) patients with spinal metastases. **Methods:** We conducted a retrospective cohort study involving 27 patients treated for CRC-derived spinal metastases at Johns Hopkins Hospital. Data on demographics, biomarker profiles of the primary colorectal tumor site, surgical outcomes, and survival were collected. Neurological function was assessed pre- and postoperatively using Frankel scores. Survival outcomes included overall survival (OS) and post-metastasis survival. **Results:** The median age of the patients was 58 years, with 63% being women. The sacral spine was the most frequently involved site (59.3%), followed by the thoracic and lumbar regions. Most patients (89%) already had extraspinal metastases, predominantly in the lungs. Biomarker analysis showed microsatellite stability in 63% of patients and CDX2 (Caudal-type homeobox 2) expression in 37%. Laminectomy was performed in 85% of cases and sacrectomy in 55.6%, leading to postoperative improvements in ambulatory function and neurological status. The main indications included local recurrence of the tumor and neurological deficits attributed to the impinging tumor. The median overall survival was 4.9 years, while the median post-metastasis survival was 3.0 years. Univariable analysis revealed that CK20 expression (*p* = 0.041) and spinal tumor recurrence (*p* = 0.045) were significantly associated with shorter post-metastasis survival. **Conclusions:** This study highlights the prognostic importance of CK20 expression and spinal tumor recurrence in CRC patients diagnosed with spinal metastases. Surgical intervention significantly improved neurological outcomes, enhancing patient quality of life. Further research with larger cohorts is needed to confirm these findings and optimize treatment strategies for this challenging patient population.

## Simple Summary

Colorectal cancer can metastasize to the spine, leading to significant complications such as pain, neurological deficits, and impaired mobility. This study examines clinical factors and biological markers that influence survival in patients with spinal metastasis from colorectal cancer. Analysis of demographics, tumor markers, surgical outcomes, and survival revealed that CK20 expression and the recurrent spinal tumors may be linked to shorter post-metastasis survival. Additionally, surgical treatment was associated with improved neurological function, enhancing patients’ quality of life. These insights could help guide future treatment strategies and improve patient care. Further prospective research with larger patient cohorts is necessary to validate these results and refine approaches for managing spinal metastases from colorectal cancer.

## 1. Introduction

Colorectal cancer (CRC) carries a serious health burden, as it is the third most commonly diagnosed cancer in men and women in the United States [1]. In 2023, approximately 153,020 new CRC cases and 52,550 related deaths were reported in the U.S., according to the American Cancer Society [2]. While CRC frequently metastasizes to the liver, lungs, and peritoneum, spinal metastases occur in 2–5% of cases, predominantly affecting men over 60 years of age [3,4].

Among cases of spinal metastasis, the thoracic spine is most commonly affected, followed by the lumbar and cervical regions [5]. Prognosis for CRC patients with spinal metastases remains poor, with a median survival of less than one year, underscoring the urgent need for early detection and effective therapeutic strategies to improve outcomes [6].

Biomarkers have become indispensable in diagnosing, monitoring, and predicting the progression of CRC. Widely studied markers, such as microsatellite stability (MSS), KRAS, CDX2 (Caudal-type homeobox 2), and CK20, are essential in CRC diagnosis and therapeutic planning [7,8,9]. These biomarkers both facilitate early detection and provide valuable insights into tumor aggressiveness, enabling the identification of high-risk subtypes and informing personalized treatment approaches. Additionally, biomarker analysis enhances prognostic accuracy and predicts treatment responses, making it a cornerstone in managing advanced CRC cases, particularly those with spinal metastases [10].

This study evaluates the clinical characteristics, oncology biomarkers, and survival outcomes in patients who underwent surgical intervention for CRC metastasis to the spine. It aims to identify the survival predictors in this cohort, offering valuable insights to guide clinical decision-making and optimize management strategies for CRC patients with metastases to the spine.

## 2. Materials and Methods

### 2.1. Study Design and Ethical Considerations

This retrospective study screened all adult patients at Johns Hopkins Medical Institutions between 2016 and 2024 with a diagnosis of CRC and confirmed spinal metastases who underwent spine-directed surgery as part of their management. Patients were excluded if they did not undergo surgery, lacked a minimum 3-month follow-up via formal office visits, or had no tumor pathology report with biomarker data. Institutional review board (IRB) approval was obtained (IRB00378753), and the study was deemed exempt from requiring participant consent due to its retrospective design. The study adhered to the ethical principles outlined in the 1975 Declaration of Helsinki, as revised in 2008, and subsequent amendments or comparable ethical standards. The manuscript follows the STROBE (Strengthening the Reporting of Observational Studies in Epidemiology) guidelines for observational studies [11].

### 2.2. Data Extraction and Outcomes of Interest

Data were extracted from medical records, including patient demographics, smoking history, metastatic spine levels, extraspinal metastases, surgical details, neurological outcomes, and treatment modalities (radiotherapy, chemotherapy, immunotherapy, and targeted therapy). Biomarker information from the primary colorectal tumor, including immunohistochemistry and genetic mutations, was also recorded. The biomarkers were identified using Multiplex PCR. Neurological outcomes were assessed by changes in Frankel scores (Grades A–E) and ambulatory status pre- and post-surgery. The Frankel scale was used, as it was the primary neurological assessment documented in the clinical records for this cohort. Although the ASIA Impairment Scale offers a more detailed evaluation, it was not consistently available across patients in this retrospective analysis.

The primary outcomes included overall survival, progression-free survival, post-metastasis survival, survival following spinal metastasis confirmation, survival after surgical resection, and postoperative neurological outcomes measured by Frankel scores. Overall survival and time to spinal metastasis were measured from the initial CRC diagnosis to death or metastasis confirmation. Progression-free survival was recorded and defined as the length of time from surgical resection of the spine tumor to cancer recurrence or death. Local recurrence was defined as recurrence at the site of surgical resection. The post-metastasis survival was defined as the duration from first imaging confirmation of spinal metastasis to death, and survival following spinal resection as the period from resection to death.

### 2.3. Statistical Analysis

Data collection was conducted in Microsoft Excel (Microsoft Corp., Redmond, WA, USA), with statistical analysis performed using IBM SPSS version 29 (IBM, Armonk, NY, USA) and R Studio 4.3.0 (RStudio Inc., Boston, MA, USA). Continuous variables were reported as medians with interquartile ranges (IQR), and categorical variables as frequencies and percentages. Kaplan–Meier (KM) curves were used to evaluate the post-metastasis survival, with patients alive at the last follow-up censored. Statistical significance was set at a *p*-value of <0.05, and univariable log-rank tests were conducted to evaluate the effect of factors on post-metastasis survival, such as tumor recurrence, CK20 positivity, age, sex, and postoperative complications.

## 3. Results

### 3.1. Patient Demographics and Clinical Characteristics

This study included 27 patients diagnosed with CRC who had metastasized to the spine and who underwent surgical management. The median age of the studied patients was 58 years (IQR: 51.4–66), and the majority were female (63%). Regarding smoking history, 55.6% of patients had never smoked, while 44.4% were ex-smokers, with no current smokers reported. The sacral spine was the most frequently affected region (59.3%), followed by the thoracic (18.5%), lumbar (18.5%), and cervical (3.7%) regions. Most patients (88.9%) had extraspinal metastases, with the lungs being the most common site (48.1%). Additional metastatic sites included the liver (33.3%), other osseous lesions (14.8%), lymph nodes (11.1%), pleura (7.4%), muscles (7.4%), kidneys (3.7%), and adrenal glands (3.7%). A total of 48.1% of patients presented with local regrowth of colorectal cancer directly to the spine, and 51.9% presented with distant metastasis to the spine (Table 1).

### 3.2. Biomarker Analysis

**Figure 1:**
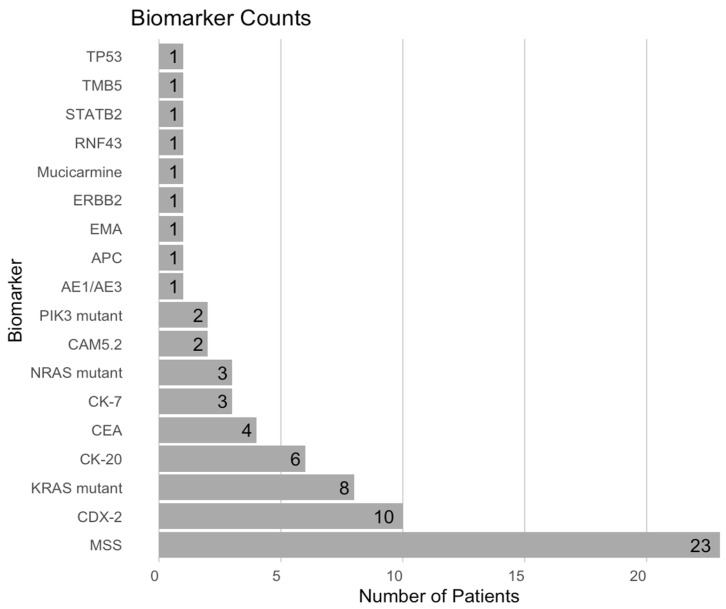
Frequency of oncology biomarkers among colorectal cancer patients with spinal metastasis.

Review of sequencing reports identified 18 unique markers through immunohistochemistry and genetic testing of the primary colorectal tumor (Figure 1). The most prevalent biomarkers included the microsatellite stability biomarker (MSS) in 23 patients (85.2%) with microsatellite instability in 1 patient (3.7%), CDX2 expression in 10 patients (37%), KRAS mutations in 8 patients (29.6%), and CK20 expression in 6 patients (22.2%). Less common markers included NRAS mutation (11.1%), CAM5.2 (7.4%), PIK3 mutation (7.4%), and several others that were each present in a single patient (3.7%), such as TP53, TMB5, STATB2, RNF43, ERBB2, EMA, and APC.

### 3.3. Surgical and Adjuvant Treatments

All patients underwent surgical treatment, with laminectomy in 85.2% and spinal fusion in 59.3%. Vertebroplasty was conducted in 22.2% of patients, and separation surgery (decompression with adjuvant radiotherapy) occurred in 7.4%. (*n* = 2) Preoperative biopsies were obtained using colonoscopy in 96.3% of cases. Staged surgeries were performed in 59.3% of cases, primarily via a posterior approach (92.6%), with the remainder using an anterior approach (7.4%). Postoperative complications occurred in 25.9% of patients, including wound breakdown (10.7%), infections (10.7%), and hematomas (3.7%). Due to the small number of complication events, statistical analysis was not powered to assess the independent impact of each complication on postoperative neurological recovery or overall survival. One patient required an immediate posterior thoracic spine epidural hematoma evacuation following surgery. Complications were more common in patients with sacral tumors (85.7%) compared to those with mobile spine tumors (14.3%) (*p* = 0.077). Most patients (66.7%) received neoadjuvant radiotherapy alone, while 14.8% underwent adjuvant radiotherapy alone and 18.5% underwent both adjuvant and neoadjuvant radiotherapy. A total of 29.6% of patients underwent stereotactic body radiation therapy (SBRT), and 70.3% received conventional radiotherapy. Immunotherapy was administered to 40.7% of patients and targeted therapy to 22.2%. Specific chemotherapy regimens included FOLFOX (44.4%), Capecitabine (48.1%), FOLFIRI (37.0%), and Oxaliplatin (25.9%). Immunotherapy agents included Nivolumab (25.9%), Bevacizumab (14.8%), anti-CTLA4 probody (7.4%), Panitumumab (7.4%), Cetuximab (3.7%), Trastuzumab (3.7%), and Pembrolizumab (3.7%), with some patients receiving dual checkpoint inhibition (11.1%).

### 3.4. Neurological and Functional Outcomes

Ambulatory function improved following surgery, with 24 (88.9%) patients being ambulatory preoperatively and 26 (96.3%) postoperatively. Neurological function, assessed using the Frankel grading system, also improved significantly. The proportion of patients with Grade E increased from 13 (48.1%) preoperatively to 18 (66.7%) postoperatively. The number of Grade D patients decreased from 11 (40.7%) to 6 (22.2%), while Grade C remained stable at 3 (11.1%).

### 3.5. Survival Analysis

The median overall survival for the cohort was 4.9 years (IQR: 3.6–6.8 years). The median time from the initial CRC diagnosis to spinal metastasis was 3.7 years (IQR: 0.7–5.9 years), and the median survival duration following spinal metastasis was 3.0 years (IQR: 1.3–4.2 years).

**Figure 2:**
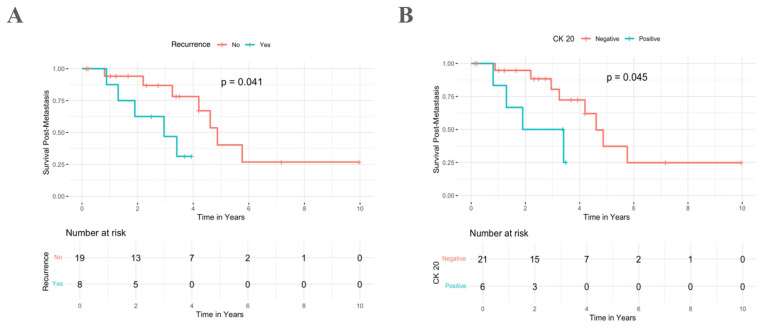
Kaplan–Meier survival analysis of the impact of (**A**) local recurrence and (**B**) CK20 expression on post-metastasis survival in patients with spinal chordoma.

Following spinal resection, the median survival was 0.8 years (IQR: 0.2–1.6 years). Progression-free survival rates were 70.4% at 2 years, 37% at 5 years, and 3.7% at 10 years for the surgical/irradiated levels. Univariable analysis indicated that local spinal tumor recurrence was significantly associated with reduced post-metastasis survival, with a median of 3.0 years for those with recurrence compared to 4.9 years for those without (*p* = 0.041) (Figure 2A). Additionally, CK20 expression is correlated with reduced post-metastasis survival, with a median survival of 1.9 years for CK20-positive patients vs. 4.6 years for CK20-negative patients; *p* = 0.045) (Figure 2B). 

Other factors, including age at treatment, sex, spinal metastatic level, lung or liver metastases, preoperative and postoperative Frankel scores, the use of immunotherapy or targeted therapy, postoperative complications, MSS status, CDX2 expression, and KRAS mutation status, did not show significant associations with survival outcomes (Table 2).

## 4. Discussion

This study enhances our understanding of the surgical outcomes, survival rates, and their association with oncology biomarkers in patients with spinal metastases originating from CRC. Most of our patients had extraspinal metastases, predominantly in the lungs, and the use of adjuvant therapies was common, including a combination of radiotherapy and chemotherapy. Neurological function improved significantly, with Frankel Grade E patients increasing postoperatively from 48.1% to 66.7%. The median overall survival was 4.9 years, with a median of 3.0 years following spinal metastasis. Recurrence and CK20 expression were significantly associated with reduced survival, highlighting their prognostic value.

The sacral spine was the most frequently affected site, followed by the thoracic and lumbar regions. In contrast to our findings, Assi et al. reported a higher occurrence of spinal metastatic infiltrations in the thoracic and lumbar regions. This discrepancy may be attributed to the valveless venous plexus of the sacrum and its proximity to pelvic organs, which facilitate the bidirectional hematogenous spread of colorectal carcinomas [12]. Additionally, approximately 90% of patients presented with extra-vertebral metastases, primarily involving the lungs. Given the propensity for multi-organ involvement in late-stage CRC, systemic imaging is essential for assessing the extent of metastases and informing treatment options and prognosis [13]. While extraspinal metastases were common in this cohort, the study was designed to focus on spinal disease-specific prognostic factors and surgical outcomes. Detailed analysis of systemic metastatic burden was not feasible due to variable documentation and was beyond the intended scope of this investigation.

In terms of treatment, our study found that partial corpectomy and laminectomy with spinal fusion significantly improved neurological outcomes and quality of life. A retrospective review by Roser et al. similarly demonstrated the efficacy of vertebrectomy and spinal fusion in achieving neural decompression and stabilization in patients with spinal metastases [14]. Despite a postoperative complication rate of 25.9% in our study, this rate aligns with prior literature, highlighting the importance of effective postoperative management [14,15]. Our findings also indicated that spinal tumor recurrence was significantly associated with reduced post-metastasis survival. Ryuk et al. noted that early recurrence within 2 years after surgery correlates with poor survival outcomes following CRC recurrence [16]. Therefore, aggressive surgical treatment may be necessary to improve survival. Similarly, a study by Chen et al. illustrated significant survival increases following extensive surgical management of spinal malignancies [17]. Most patients in our study received radiotherapy and chemotherapy for treatment.

Joharatnam-Hogan et al. revealed that multimodal treatment (radiotherapy, chemotherapy, immunotherapy, and surgery) resulted in significantly higher survival outcomes than chemotherapy alone [18]. These findings underscore the importance of comprehensive treatment approaches for improving outcomes in CRC patients with spinal metastasis.

Regarding survival outcomes specifically, our cohort demonstrated a median overall survival of 4.9 years; however, this decreased to 3 years following spinal metastasis and further declined to 0.8 years after surgical resection. Bostel et al. demonstrated similar findings, reporting a median survival of only 4.2 months for patients undergoing radiotherapy for spinal metastasis from CRC [6]. Additionally, while spinal tumor recurrence was significantly associated with reduced post-metastasis survival, Alamanda et al. found that revision surgery for metastatic spinal tumor recurrence did not correlate with decreased survival outcomes, suggesting that multiple factors may influence these results [19]. Notably, ambulatory and neurologic function significantly improved following surgery; Liu et al. similarly described significant improvements in ambulatory function for patients undergoing surgery for spinal metastasis [20].

In our cohort, MSS (microsatellite stability), CDX2 loss, KRAS mutations, and CK20 expression were identified as the most common biomarkers relevant to metastatic CRC diagnosis and prognosis. Our results indicated that CK20 expression was linked to reduced survival; similarly, Ning et al. found that high CK20 expression significantly correlated with worse survival outcomes than low expression levels, which may be due to its role in tumor differentiation and association with aggressive tumor phenotypes [21,22]. Although CK20 is not independently predictive, its combination with CDX2 enhances diagnostic precision and supports prognostic assessments when identifying sources of metastases [23,24]. By linking CK20 expression to tumor subtypes in CRC, this dual-marker strategy may help clinicians optimize individualized therapy strategies and improve patient outcomes [23,24]. Recent advancements have focused on MSS as a genetic marker, indicating deficiencies in DNA mismatch repair mechanisms; high MSS (MSS-H) tumors exhibit increased sensitivity to immunotherapies, such as pembrolizumab and nivolumab, which enhance immune detection and response against cancer cells [25,26,27,28]. Identifying MSS status is crucial for personalizing CRC treatment strategies. CDX2—a biomarker associated with tumor differentiation—also holds significant clinical relevance in CRC management; it is typically expressed in healthy colon cells but is often absent in poorly differentiated CRCs, correlating with aggressive disease and poorer outcomes. While CK20 expression was significantly associated with recurrence, other biomarkers, such as CDX2, MSS, and KRAS, did not reach statistical significance. This may reflect limited statistical power and biological heterogeneity rather than a true lack of prognostic relevance.

Despite the potential significance of these biomarkers in CRC management, MSS status, CDX2 expression, and KRAS mutation status were not significantly associated with survival outcomes in our cohort. In contrast, Slik et al. concluded that loss of CDX2 in MSS patient groups predicted adverse clinical outcomes for CRC patients [29]. Additionally, Popat et al. indicated that CRC patients exhibiting MSS have a worse prognosis compared to those with microsatellite instability (MSI), contrasting with our current findings [30]. The prognostic value of KRAS mutations remains debated; while some studies, like Arrington et al., suggest an unfavorable prognosis associated with KRAS mutations in late-stage CRCs, other studies, including our own, have not found a significant association [31]. Further research is necessary to determine the relevance of such nonsignificant findings to CRC treatment. Additionally, this study is limited by its small sample size, retrospective single-institution design, and the exclusion of non-surgical patients. These factors introduce potential selection bias and limit the generalizability of the findings. Similarly, the statistical analysis was limited to univariable models due to the small sample size. The inability to perform multivariable analysis restricts the ability to control for potential confounding factors and limits the strength of causal inferences for factors such as CK20 and other biomarkers. Further validation in larger, prospective, and multicenter cohorts is warranted.

###  Study Limitations and Future Directions

This study is limited by its retrospective design, which may introduce biases in patient selection and data collection. The exclusion of patients who did not undergo surgery or lacked sufficient follow-up could skew findings toward individuals with more favorable prognoses. Furthermore, the small sample size and single-institution scope reduce statistical power and may not reflect the full heterogeneity of CRC patients with spinal metastases. Future research should address these limitations through multicenter, prospective studies involving larger and more diverse patient cohorts to enhance generalizability. Additionally, long-term studies evaluating the impact of surgical techniques, adjuvant therapies, and the timing and sequencing of immunotherapy and targeted treatments are essential. Such efforts will refine survival outcomes, reduce complications, and ultimately improve the quality of life for CRC patients with spinal metastases.

## 5. Conclusions

Our study highlights the prognostic importance of CK20 expression and tumor recurrence on survival in CRC patients with spinal metastases. Surgical intervention notably improved neurological function and ambulatory status, highlighting its role in enhancing patient quality of life. While these findings provide valuable insights into the clinical management of spinal metastases in CRC, the small sample size (*n* = 27) limits statistical power and generalizability. Further research with larger, multicenter cohorts is needed to confirm these associations and refine treatment strategies, particularly for emerging biomarkers and personalized therapeutic approaches. Prospective, multi-institutional studies will also be critical to validate these findings and support the development of standardized surgical indications for this complex patient population.

## Figures and Tables

**Table 1:** Demographic and Clinical Characteristics of Study Population (*n* = 27).

Parameter	Description, *n* (%)
Age (years)	Median (IQR) = 58.0 (51.4–66.0)
Sex	M: 10 (37.0) F: 17 (63.0)
Smoking Status	Never Smoked: 15 (55.6) Ex-smokers: 12 (44.4) Current smokers: 0
Mortality Rate	15 (55.6)
Overall Survival	Median (IQR) = 4.9 (3.6–6.8)
Time to Spine Metastasis (years)	Median (IQR) = 3.7 (0.7–5.9)
Post-Metastasis Survival	Median (IQR) = 3.0 (1.3–4.2)
Post-Spinal-Metastasis Survival	Median (IQR) = 1.8 (0.3–2.6)
Post-Spinal-Resection Survival	Median (IQR) = 0.8 (0.2–1.6)
Progression Free Survival	2 years: 19 (70.4) 5 years: 10 (37.0) 10 years: 1 (3.7)
Spine Level	Cervical: 1 (3.7) Thoracic: 5 (18.5) Lumbar: 5 (18.5) Sacral: 16 (59.3)
Other Tumors	Lung: 13 (48.1) Liver: 9 (33.3) Other Bony Lesions: 4 (14.8) Lymph Node: 3 (11.1) Pleural: 2 (7.4) Muscle: 2 (7.4) Kidney: 1 (3.7) Adrenal Gland: 1 (3.7) No Metastases: 3 (11.1)
Preop Frankel Score	C: 3 (11.1) D: 11 (40.7) E: 13 (48.1)
Postop Frankel Score	C: 3 (11.1) D: 6 (22.2) E: 18 (66.7)
Preop Ambulatory Status	Ambulatory: 24 (88.9) Non-Ambulatory: 3 (11.1)
Postop Ambulatory Status	Ambulatory: 26 (96.7) Non-Ambulatory: 1 (3.7)
Procedures Performed	Pre-Op Biopsy: 26 (96.3) Laminectomy: 23 (85.2) Fusion: 16 (59.3) Staged Surgery: 16 (59.3) Vertebroplasty: 6 (22.2)
Procedure Approach	Anterior: 2 (7.4) Posterior: 25 (92.6)
Treatment	Radiotherapy: 26 (96.3) Adjuvant Neoadjuvant Chemotherapy: 26 (96.3) Folfox: 12 (44.4) Capecitabine: 13 (48.1) Folfiri: 10 (37.0) Oxaliplatin: 7 (25.9) Trifluridine/Tipiracil: 5 (18.5) Fluorouracil: 2 (7.4) Leucovorin: 1 (3.7) Epirubicin: 1 (3.7) Immunotherapy: 11 (40.7) Nivolumab: 7 (25.9) CTLA4 probody: 2 (7.4) Pembrolizumab: 1 (3.7) Double checkpoint: 1 (3.7) Cetuximab: 1 (3.7) Targeted Therapy: 6 (22.2) Bevacizumab: 4 (14.8) Denosumab: 2 (7.4) Panitumumab: 2 (7.4) Panitumumab: 2 (7.4) Copanlisib: 1 (3.7) Regorafenib: 1 (3.7) Trastuzumab: 1 (3.7)
Postop Complications	No Complications: 20 (74.1) Complications: 7 (25.9)
Spine Tumor Recurrence	8 (29.6)

**Table 2:** Univariable results derived from log rank test to evaluate the effect on survival post metastasis.

Variable	Patients *n* (%)	Survival Post-Metastasis		
		Number of Deaths	SPM (years) (Median ± SE)	*p*-Value
Age at Treatment				
<58	13 (48.1)	7/13 (53.8)	4.6 ± 0.7	0.863
≥58	14 (51.9)	5/14 (35.7)	4.9 ± 1.3	
Sex (M)				
Female	17 (63.0)	8/17 (47.1)	4.2 ± 0.7	0.758
Male	10 (37.0)	4/10 (40.0)	4.6 ± 1.4	
Spine Level Metastasis				
Cervical or Thoracic	6 (22.2)	3/6 (50.0)	4.6 ± 0.0	0.507
Lumbar	5 (18.5)	3/5 (60.0)	1.9 ± 1.3	
Sacral	16 (59.3)	6/16 (37.5)	4.2 ± 1.3	
Lung Metastasis				
No	14 (51.9)	4/14 (28.6)	3.3 ± 2.6	0.185
Yes	13 (48.1)	8/13 (61.5)	4.2 ± 1.0	
Liver Metastasis				
No	18 (66.7)	8/18 (44.4)	4.9 ± 1.0	0.264
Yes	9 (33.3)	4/9 (44.4)	4.6 ± 0.0	
Preop Frankel Score				
C or D	14 (51.9)	7/14 (50.0)	3.4 ± 0.8	0.696
E	13 (48.1)	5/13 (38.5)	4.9 ± 0.5	
Postop Frankel Score				
C or D	9 (33.3)	5/9 (55.5)	4.6 ± 1.5	0.671
E	18 (66.6)	7/18 (38.9)	4.2 ± 1.0	
Immunotherapy				
No	16 (59.3)	6/16 (37.5)	4.2 ± 1.3	0.980
Yes	11 (40.7)	6/11 (54.5)	4.6 ± 1.2	
Targeted Therapy				
No	21 (77.8)	10/21 (47.6)	6.6 ± 4.6	0.822
Yes	6 (22.2)	2/6 (33.3)	4.3 ± 0.0	
Postop Complications				
No	20 (74.1)	8/20 (40.0)	4.9 ± 1.6	0.277
Yes	7 (25.9)	4/6 (66.7)	4.2 ± 1.5	
Tumor Recurrence				
No	19 (70.4)	7/19 (36.8)	4.9 ± 0.4	0.041
Yes	8 (29.6)	5/8 (62.5)	3.0 ± 0.9	
MSS				
No	10 (37.0)	4/10 (40.0)	5.8 ± 0.0	0.467
Yes	17 (63.0)	8/17 (47.1)	4.6 ± 0.8	
CDX-2				
No	17 (63.0)	7/17 (41.2)	4.6 ± 0.5	0.055
Yes	10 (37.0)	5/10 (50.0)	2.2 ± 0.0	
CK-20				
No	21 (77.8)	8/21 (38.1)	4.6 ± 0.4	0.045
Yes	6 (22.2)	4/6 (66.7)	1.9 ± 1.0	
KRAS				
No	19 (70.4)	8/19 (42.1)	4.9 ± 1.4	0.931
Yes	8 (29.6)	4/8 (50.0)	4.2 ± 1.0	

## Data Availability

The data presented in this study are available on reasonable request from the corresponding author. The data are not publicly available due to privacy and institutional data-sharing restrictions.
